# Challenges in the management of metastatic gastrointestinal stromal tumor in a patient with neurofibromatosis type 1: a case report

**DOI:** 10.1186/s13256-022-03382-y

**Published:** 2022-05-01

**Authors:** B. G. Bharath, Sameer Rastogi, Shamim Ahmed, Adarsh Barwad

**Affiliations:** 1 Department of Medical Oncology, All India Institute of Medical Sciences, New Delhi, India; 2 Department of Nuclear Medicine, All India Institute of Medical Sciences, New Delhi, India; 3 Department of Pathology, All India Institute of Medical Sciences, New Delhi, India

**Keywords:** Gastrointestinal stromal tumor (GIST), Neurofibromatosis type 1 (NF 1)

## Abstract

**Background:**

Neurofibromatosis type 1 is an inherited cancer predisposition syndrome that is caused by a mutation in the *NF1* gene that encodes neurofibromin. Patients with neurofibromatosis type 1 have a higher risk of gastrointestinal stromal tumor. This study reports the case of a patient with gastrointestinal stromal tumor who was later diagnosed to have neurofibromatosis type 1 and, unlike usual features, had some uncommon features such as occurrence at an early age and unusual site of origin.

**Case:**

We report the case of a 29-year-old Indian female diagnosed to have gastrointestinal stromal tumor originating from the greater curvature of the stomach. Gastrointestinal stromal tumor was wild type, negative for c-kit and platelet-derived growth factor receptor, and had an aggressive clinical course not responding to oral tyrosine kinase inhibitors. On later evaluation, we found that the patient had germline mutation in *NF1*. This case has some unusual features compared with gastrointestinal stromal tumor cases reported in neurofibromatosis type 1. Firstly, the age of onset for gastrointestinal stromal tumor in neurofibromatosis type 1 is earlier in our case compared with previous cases reported in literature. Secondly, the site of occurrence is in the stomach, without involving other parts of the intestine. Gastrointestinal stromal tumor in neurofibromatosis type 1 is usually multifocal, and small intestine is the common site of occurrence. When occurring in the stomach, it is usually associated with other lesions in the small intestine. Lastly, the clinical course is aggressive compared with previous case reports and series.

**Conclusion:**

Our patient had germline *NF1* mutation and cutaneous stigmata of neurofibromatosis. Our patient had unicentric gastrointestinal stromal tumor occurring at younger age and involving greater curvature of the stomach, with spindle cell type histology and high-risk features. If gastrointestinal stromal tumor occurs at young age, we should look into neurocutaneous markers.

## Introduction

GIST is the most common mesenchymal neoplasm affecting the gastrointestinal (GI) tract. About 80% of GISTs have mutations in the *KIT* proto-oncogene that lead to constitutive activation of KIT, a receptor tyrosine kinase (RTK) [1]. A subset of GISTs lacking *KIT* gene mutations harbor activating mutations in a related RTK, platelet-derived growth factor receptor-alpha (PDGFRA). Approximately 12% of GISTs have no mutation in either *KIT* or *PDGFRA*. The majority of these GISTs have mutations or epigenetic silencing of succinate dehydrogenase (SDH) subunits leading to SDH-deficient GIST.

GISTs associated with NF1 syndrome are rare, accounting for only 6–7% of all GIST cases [2]. Neurofibromatosis type 1 (NF-1) is an autosomal-dominant genetic disorder with nearly 100% penetrance [3]. Neurofibromatosis results from loss-of-function mutation in *NF1* gene on chromosome 17q21. Loss of NF1 leads to unopposed activation of the RAS pathway and to abnormal proliferation and survival of cells [4]. Therefore, patients with NF1 have a higher risk of developing both benign and malignant tumors. Malignant peripheral nerve sheath tumor is the most common tumor in patients with NF1. Other malignant tumors strongly associated with NF1 include rhabdomyosarcoma, gastrointestinal stromal tumors, neuroectodermal tumors, pheochromocytomas, and breast carcinoma.

In patients with NF1, there is about a 45-fold increased risk for developing gastrointestinal stromal tumors (GIST) compared with sporadic GIST. GIST is diagnosed typically at earlier age (almost a decade earlier) among NF1 patients than those with KIT/PDGFRA-mutated GISTs [5]. Secondly, GIST in NF1 predominantly affects young females and commonly involves the small bowel [6]. GIST occurring in patients with NF1 will have no activation mutations in KIT (exons 9, 11, 13, 17), PDGFRA (exons 12, 14, 18), and BRAF. Hence, GIST in patients with NF1 responds poorly to TKIs such as imatinib, sunitinib, or regorafenib. We report herein the case of a 29-year-old female who had NF1 whose diagnosis was established after occurrence of GIST. She had unique features such as GIST occurring at relatively younger age, unifocal GIST, and unusual site of occurrence.

## Case report

**Fig. 1:**
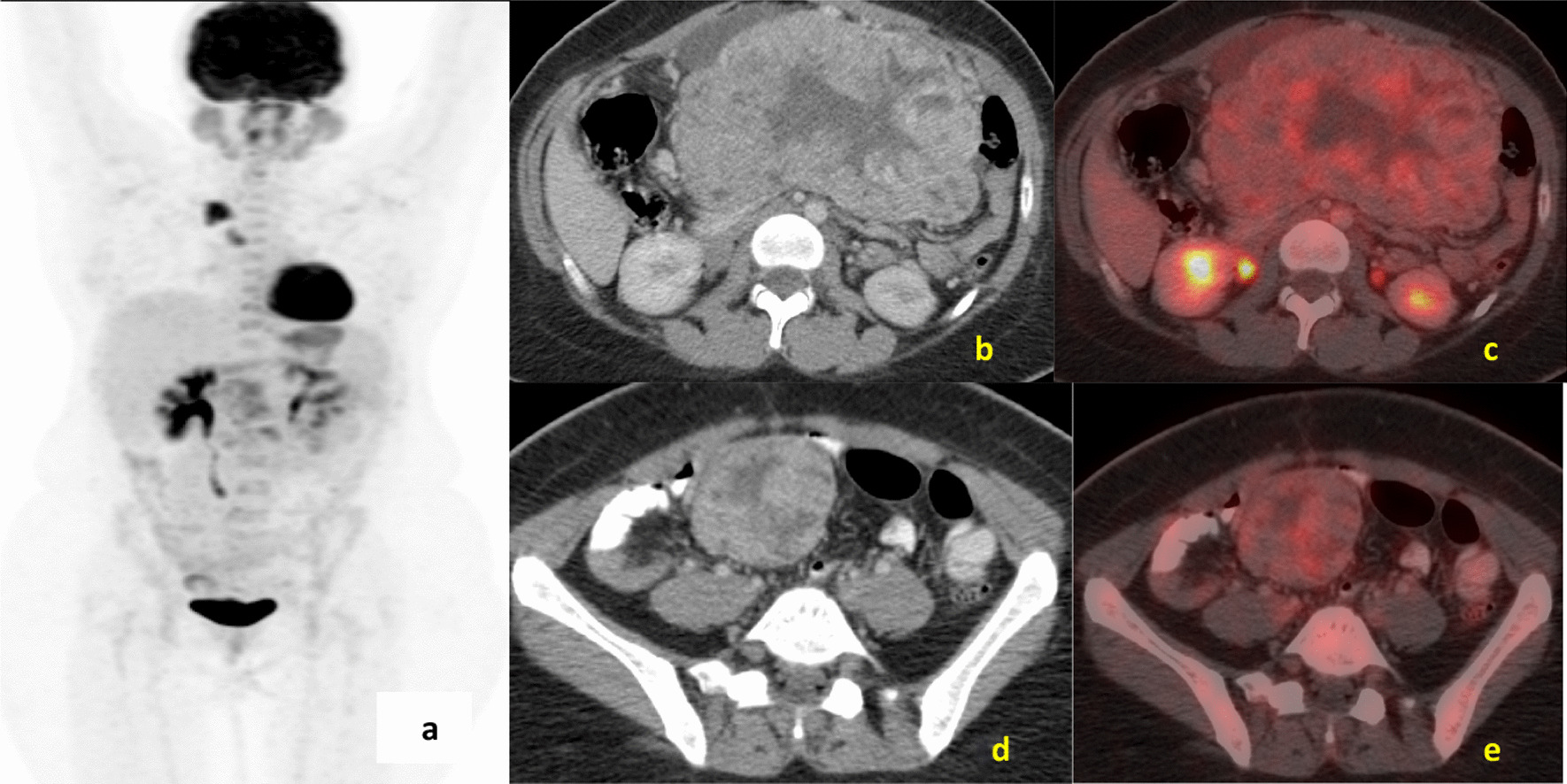
**a** Maximum-intensity projection image of fluorodeoxyglucose positron emission tomography - computerized tomography, showing heterogeneous tracer uptake in the abdominal region corresponding to a large heterogeneous mass in the mesentery on axial computerized tomography section (**b**) showing increased uptake on fused fluorodeoxyglucose positron emission tomography - computerized tomography (**c**). **d** Axial computerized tomography scans of pelvis region showing heterogeneous mass in the pelvic cavity. Increased uptake is present in fused fluorodeoxyglucose positron emission tomography - computerized tomography image (**e**)

**Fig. 2:**
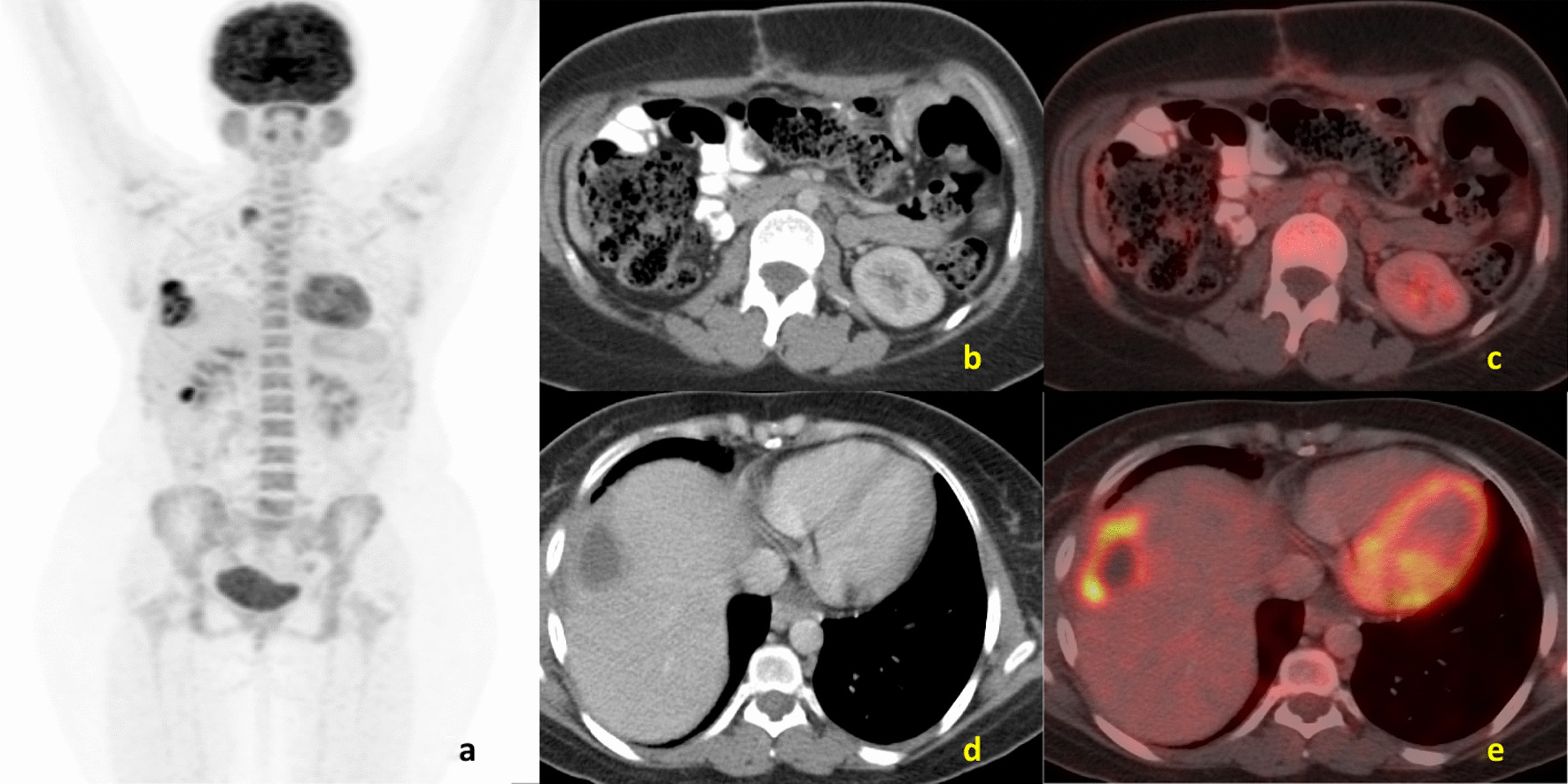
**a** Maximum-intensity projection image of fluorodeoxyglucose positron emission tomography - computerized tomography showing increased tracer uptake in the right upper abdominal region corresponding to a large heterogeneous lesion in segment IVa/VIII of liver on axial computerized tomography section (**d**) showing increased uptake on fused fluorodeoxyglucose positron emission tomography - computerized tomography (**e**) suggestive of liver metastasis. **b** Axial computerized tomography of abdomen showing postsurgical changes with excision of the mesenteric mass and no abnormal focal uptake on fused fluorodeoxyglucose positron emission tomography - computerized tomography image (**c**)

**Fig. 3:**
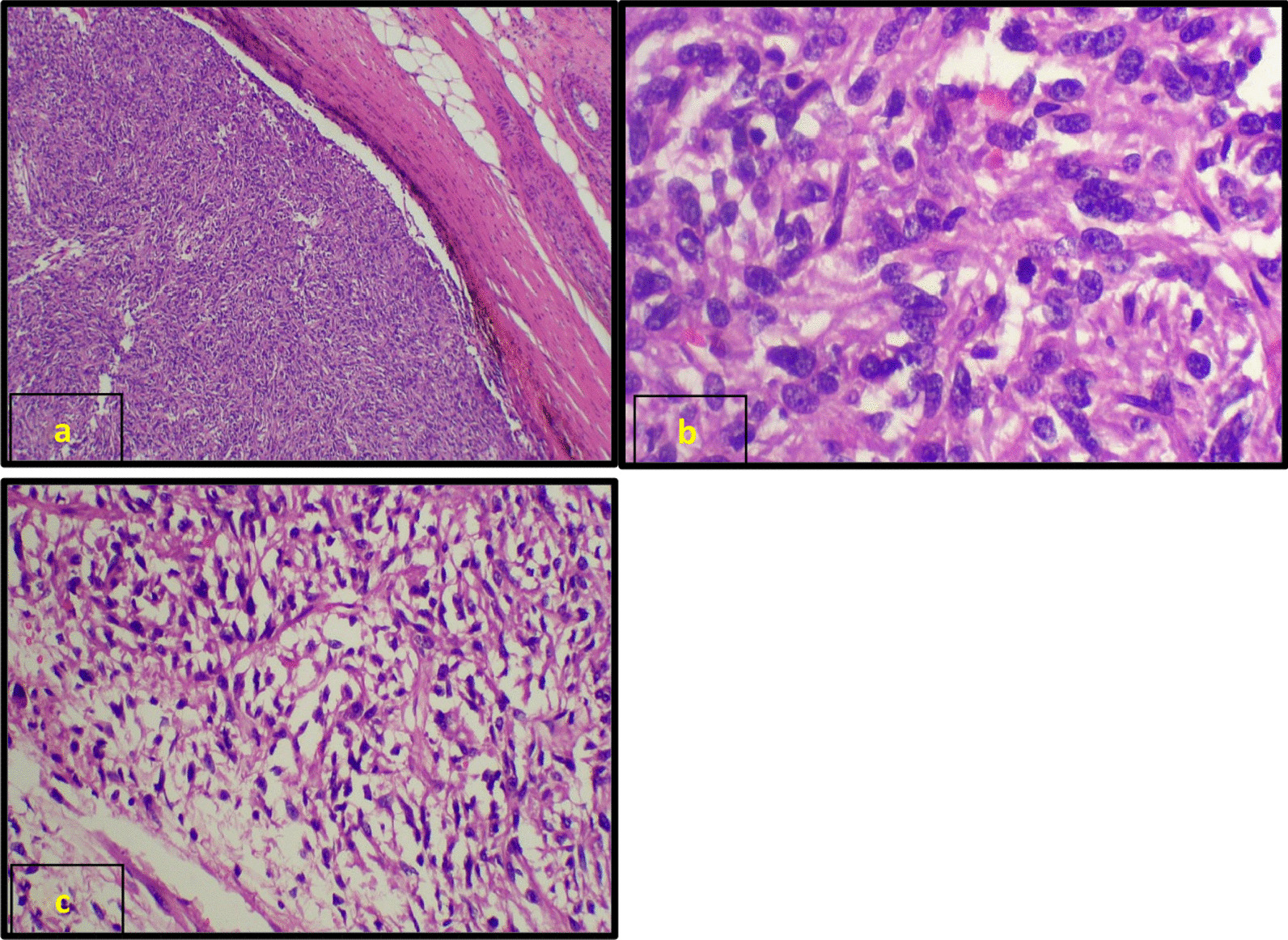
**a** Section from gastric mass showing neoplastic spindle cells in fascicles within muscularis propria. **b** Neoplastic spindle cells with significant atypia and mitosis (arrow). **c** Significant epithelioid change (20%) was evident with abundant pale to clear cytoplasm

**Fig. 4:**
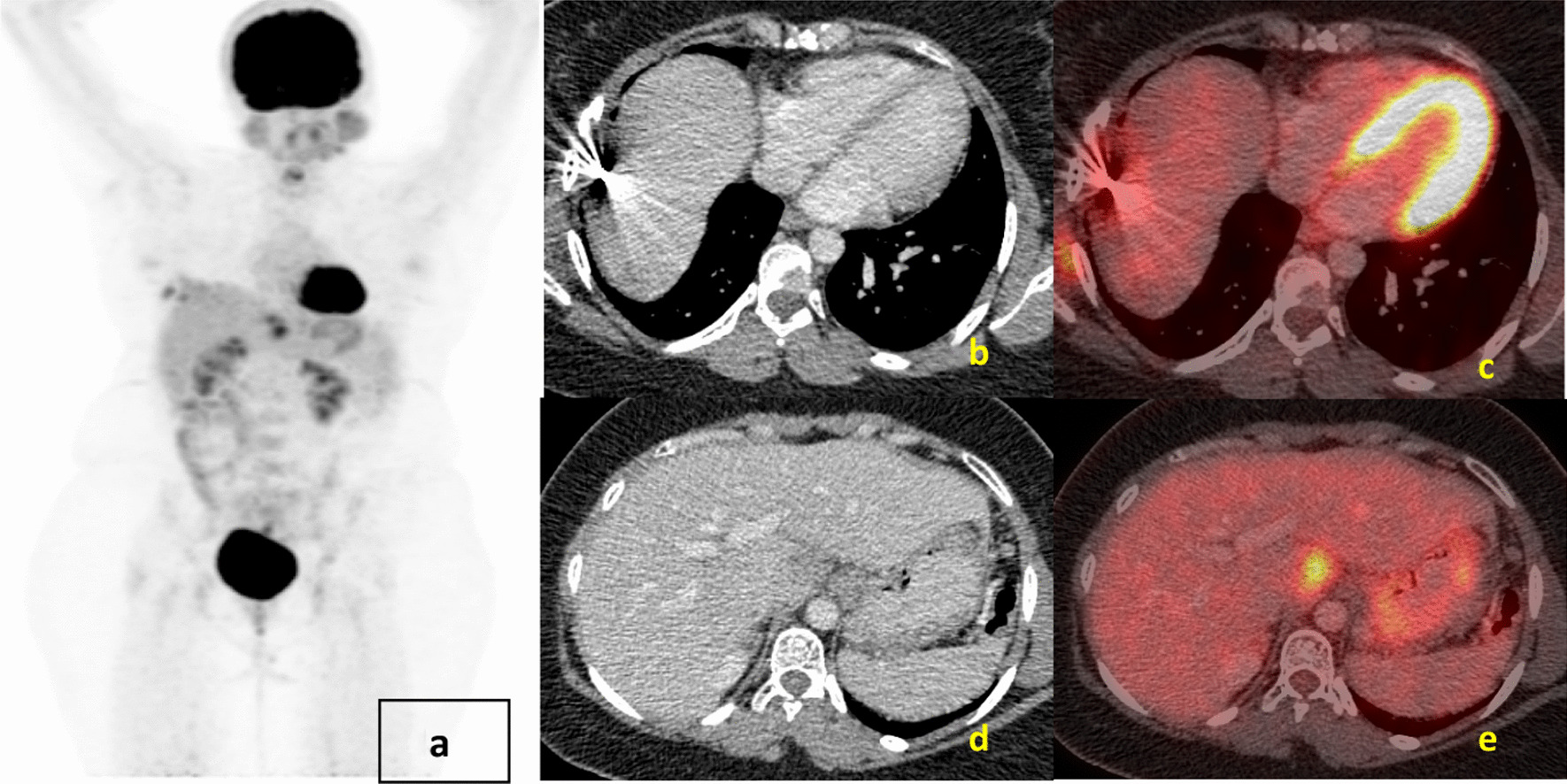
**a** Maximum-intensity projection image of fluorodeoxyglucose positron emission tomography - computerized tomography showing increased tracer uptake in the right upper abdominal region corresponding to artifactual uptake (**c**) in site of previously seen metastasis of liver (**b**). **d** Axial CT abdomen region showing hypodense lesion in caudate lobe of liver showing uptake on fused fluorodeoxyglucose positron emission tomography - computerized tomography image (**e**)

A 29-year-old female of Indian origin with no prior comorbidities was diagnosed with metastatic GIST in March 2019. She had nausea and loss of appetite for 2 weeks in March 2019. She underwent initial evaluation at an outside hospital for her symptoms. Upper GI endoscopy showed relaxed lower esophageal sphincter and poorly distensible stomach, with external mass effect. Whole-body positron emission tomography (PET)/computed tomography (CT) done in March 2019 (Fig. 1) showed a soft tissue mass lesion in the left hypochondrium abutting the greater curvature of the stomach and measuring 14.2 × 15.1 × 17.1 cm^3^ with hypodense lesion in the liver. CT-guided biopsy showed poorly differentiated malignant round cell tumor, and immunohistochemistry (IHC) was positive for CD117, CD34, and DOG1, suggestive of gastrointestinal stromal tumor. The tumor had a low Ki-67 index of 2%, and C-KIT mutation analysis showed exon 9 and exon 11 wild type. She was started on imatinib 400 mg OD, which she took for 3 months before disease progression. PET/CT scan done at progression in June 2019 (Fig. 2) showed heterogeneously enhancing mass lesion in the gastrocolic ligament, mesentery, and omentum, possibly arising from the inferior surface of the greater curvature/body–antrum of the stomach with peritumoral, intratumoral vascularity, and mild ascites. There were multiple liver metastatic lesions, increased in number compared with baseline imaging. She underwent laparoscopic subtotal gastrectomy and liver metastasectomy in June 2019. Postoperative histopathological examination (Fig. 3) confirmed gastrointestinal stromal tumor of predominantly spindle type (80%), grade 2, high risk, with free margins, and about 10% necrosis was present in the tumor. Follow-up scan done 2 months after resection in August 2019 (Fig. 4) showed residual heterogeneously enhancing mass lesion in the gastrocolic ligament, mesentery, and omentum. There was interval development of new enhancing lesions in segments VI, II, VII, and IVA of the liver. There was also interval development of peripherally enhancing hypodense lesion in segment VIII of the liver in subcapsular location. The patient was treated with fluorodeoxyglucose (FDG) PET and magnetic resonance imaging (MRI)-based stereotactic body radiotherapy with CyberKnife technique to liver lesions with a dose of 35 Gy in five fractions and lung and mediastinal lymph nodes to a dose of 30 Gy in five fractions. She was started on sunitinib 50 mg OD for 2 weeks every 3 weeks.

**Fig. 5:**
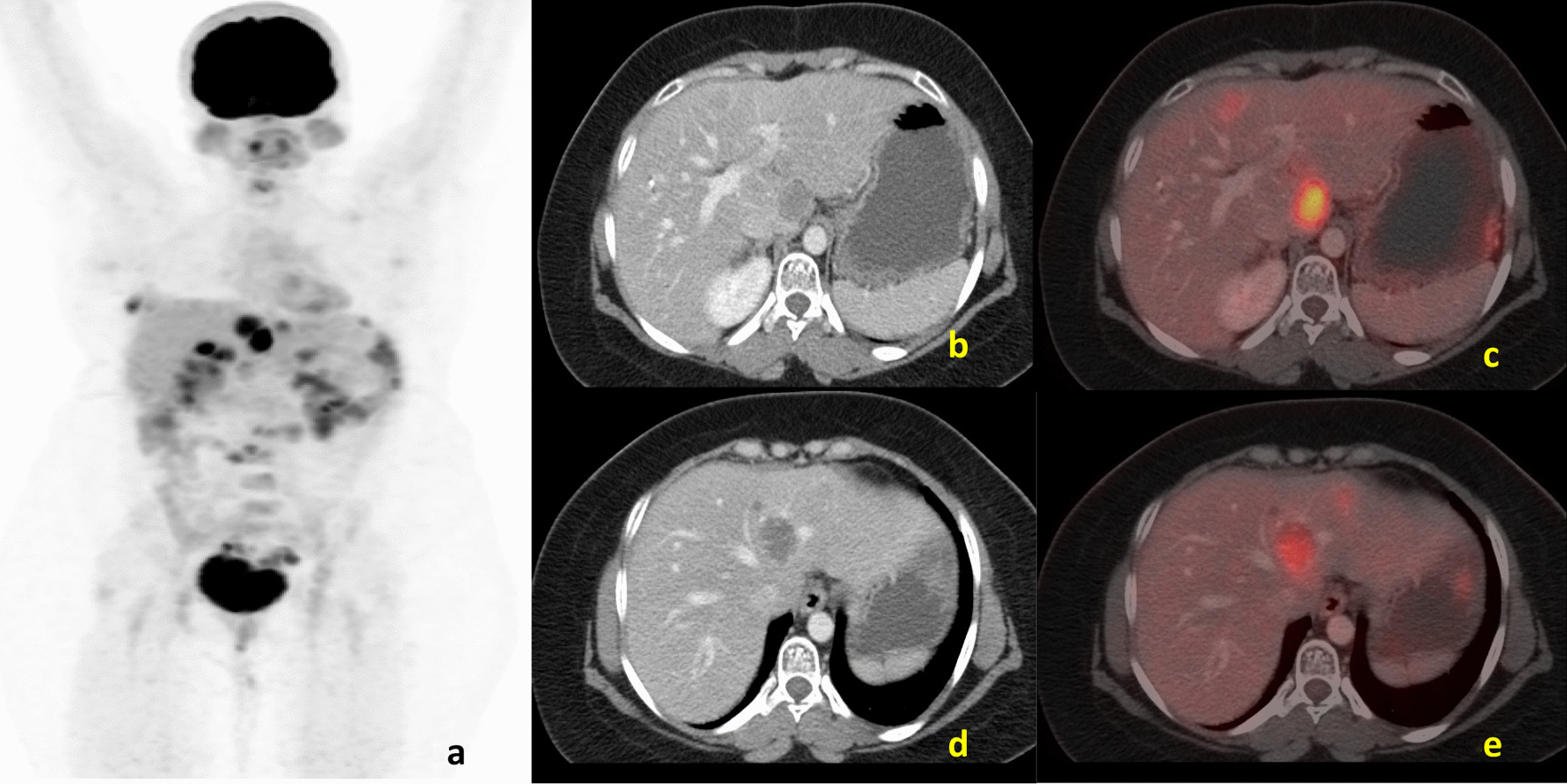
**a** Maximum-intensity projection image of fluorodeoxyglucose positron emission tomography - computerized tomography showing increased tracer uptake in the right upper abdominal region corresponding to multiple liver lesions on (**b**, **d**) showing increased uptake in the fused fluorodeoxyglucose positron emission tomography - computerized tomography images (**c**, **e**) suggestive of progressive disease compared with previous scan

Follow-up fluorodeoxyglucose positron emission tomography - computerized tomography in March 2020 showed interval regression of liver lesions. However, there was a new osteolytic lesion involving the right scapula, not associated with any soft tissue component. She received radiotherapy to the scapular lesion, and sunitinib was continued. The latest fluorodeoxyglucose positron emission tomography - computerized tomography scan done in January 2021 (Fig. 5) showed evidence of interval appearance of multiple hypermetabolic lesions in the liver, omentum, and peritoneum along with mesenteric lymphadenopathy. The focal hypermetabolic lesion in the seventh rib was present, and there was a stable scapular lesion. Overall features were suggestive of progressive disease.

**Fig. 6:**
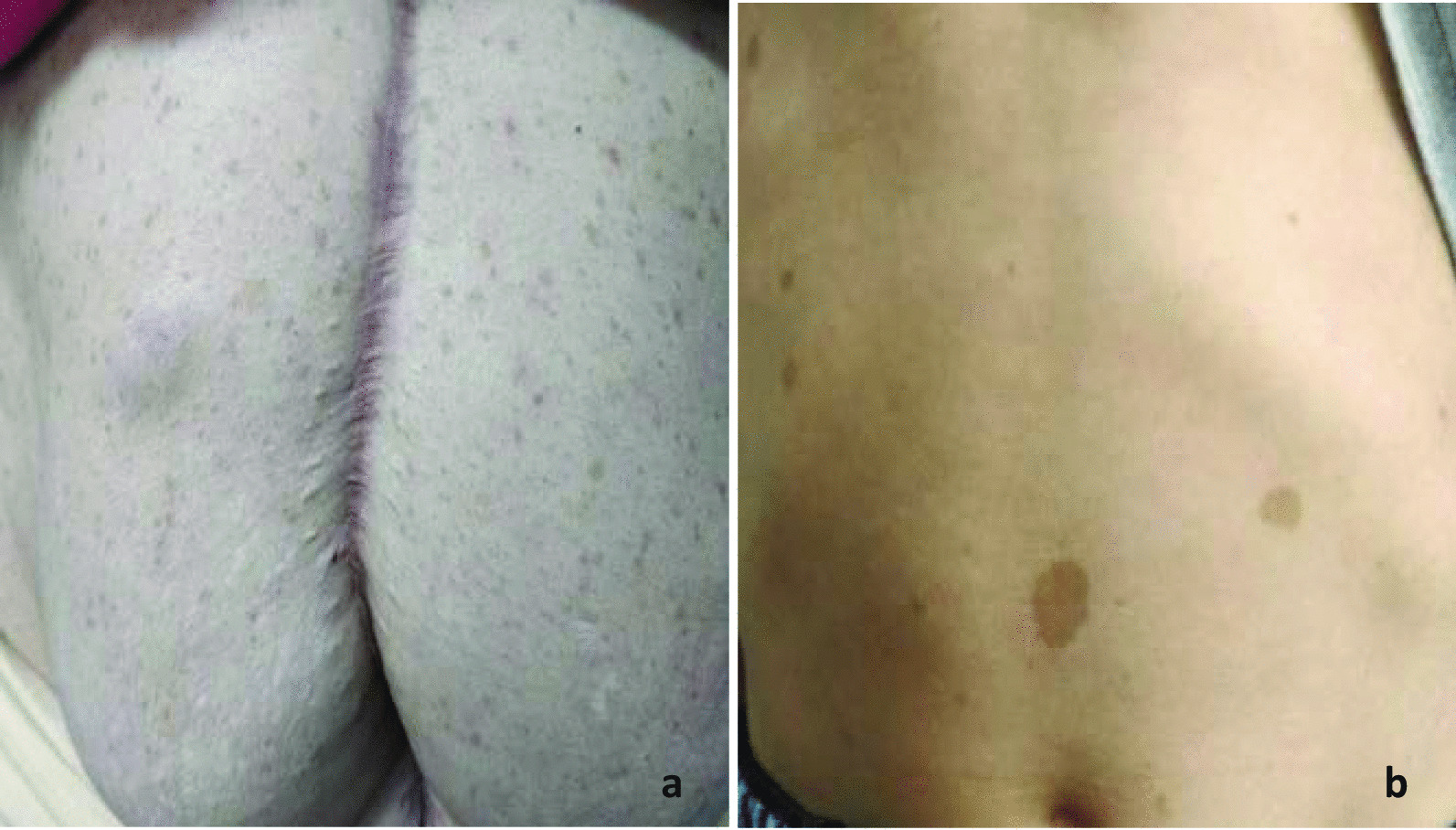
**a** Multiple café-au-lait macules present all over the trunk. **b** Patient’s child, who is 4 years of age, also with café-au-lait macules

**Fig. 7:**
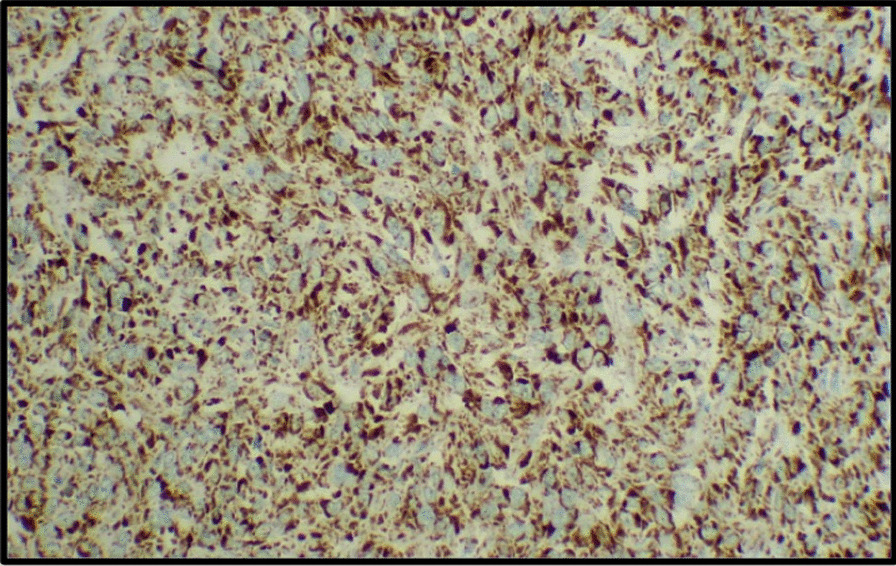
SDH-B immunoexpression was retained throughout the tumor

She was referred to us for further evaluation. On thorough examination, we found multiple café-au-lait macules present all over the trunk (Fig. 6). She had axillary frecklings. She did not have optic nerve glioma, Lisch nodules, or plexiform neurofibromas. Her child, who was 4 years of age, also had café-au-lait macules. Her parents did not have features suggestive of neurofibromatosis 1. Her examination findings were suggestive of neurofibromatosis, hence *NF1* mutational analysis was done in tumor tissue. *NF1* mutation was detected in exon 45. Histopathology review was done, and histology was confirmed. DOG1 (discovered on GIST1) and CD117 were positive in the tumor tissue. SDH-B (succinate dehydrogenase B) immunoexpression was retained throughout the tumor (Fig. 7). Based on the study by Schoffski *et al.*, we decided to treat her with cabozantinib. She was started on cabozantinib with effect from January 2021. The patient took medicine for 4 months before she had clinical progressive disease. She stopped taking cabozantinib and started using alternative medicine. She died in June 2021 due to progressive disease.

## Discussion

NF1 is the common type (> 90%) of neurofibromatosis, being seen in 1 in 2000–5000 childbirths [7]. About half of the cases are due to familial mutation, with the remaining half due to sporadic gene mutation. Even though mutation has 100% penetrance, clinical manifestation ranges from mild or subtle signs to multiple malignant tumors. Our patient was diagnosed to have NF1 when she was 29 years of age. The average age of symptom onset in NF1 is 20 years, and diagnoses are usually made by the age of 28 years [8]. The interesting feature of this case is that the diagnosis was made while evaluating for metastatic GIST. Usually, the incidence of cancer in patients with NF1 is high (2.7-fold) after 50 years of age [9], and the overall incidence of malignancy in patients with NF1 ranges between 4% and 52%. GIST is the second most common malignancy seen in NF1, with incidence of up to 25% [10]. Median age of diagnosis of sporadic GIST is 55–65 years. GIST in NF1 is diagnosed at a somewhat younger age of 50 years [5]. However, our patient developed GIST at the age of 27 years. In a known case of NF1, occurrence of abdominal symptoms such as gastrointestinal bleeding, anemia, abdominal pain, or palpable abdominal mass should stimulate investigation for GIST, even at younger age. Our patient presented with nonspecific symptoms such as early satiety.

Multicentricity is not a feature of sporadic GIST. However, GIST in NF1 patients is usually multiple and multicentric disease, hence the presence of these features in GIST should alert clinicians to evaluate for NF1. Another scenario where the patient can have GIST at multiple sites includes patients with family history of GIST and germline KIT mutations in patients with Carney’s triad and a distinctive syndrome characterized by multicentric paragangliomas and GIST. Our case was different in that the patient at diagnosis had only one tumor mass abutting the greater curvature of the stomach along with a single metastatic lesion in the liver. A study published by Bulusu *et al.* [11] included 15 patients of NF1 with GIST. Among these patients, 33% were multifocal and 67% were unifocal. The primary site of GIST among patients included in this study were stomach (6.6%), duodenum (33%), small bowel (67%), and colon (6.6%). The small bowel was the common site of involvement. This is because GIST in NF1 patients differs in its distribution pattern compared with sporadic GIST. The stomach is the common site in the case of sporadic GIST, whereas the small intestine is the common site of involvement in NF1 patients. In the study mentioned above (Bulusu *et al*.), only four (26%) patients had gastric tumors and all of them were also associated with small intestinal tumors. In contrast, our patient had a primary tumor in the stomach without involving other parts of the intestine. Another study [12] that included only duodenal GIST published data of 156 duodenal GIST patients. In that study, out of a total of 156 patients, 10 were found to have NF1, and 6 of them also had multiple GISTs in other parts of the small intestine. In another study by Anderson *et al*. [13], 26% of patients with NF1 had GIST arising in the stomach. However, in all these cases, patients had concurrent tumors at other sites of the small intestine.

There are three histologic subtypes in GIST. The most common is spindle cell type (70%), followed by epithelioid type (20%) and mixed histology. NF1-associated GIST will predominantly have spindle cell morphology (up to 80%) [14]. Our patient had mixed histology with 80% spindle cell morphology and 20% epithelioid morphology.

GIST in patients with NF1 usually has low-risk features. In a study conducted by Anderson *et al*. [13], which was a population-based series of 288 GIST patients, about one-third of patients had high-risk or overtly malignant GIST and one-half had a very low- or low-risk GIST. Their study included 15 patients who had NF1 with GIST, and none of them died due to GIST. However, our patient had high-risk features and also clinical behavior was aggressive with multiple recurrences within a short period. We mention here that GIST in patients with NF1 has an unpredictable clinical course.

NF1-associated GISTs also have a very high rate of CD34 immunoreactivity as compared with sporadic GIST. Up to 90% of NF1 patients with GIST stain positive for CD34 compared with only about half patients with sporadic GIST. Our patient had CD34 positivity in the tumor. Also, these tumors are genotypically distinct from sporadic GIST in that they lack KIT mutations. There are only two case reports in literature that describe NF1-associated GIST harboring KIT mutations. There is no literature for NF1 patients with GIST harboring PDGFRA. The absence of KIT and PDGFRA mutations was also shown in large case series such as that by Anderson *et al*. [13]. This shows that the pathogenesis of NF1-associated GIST differs from most sporadic GIST. However, irrespective of KIT mutation status, KIT receptor activation occurs ubiquitously in GIST.

From the treatment point of view, our patient had disease refractory to tyrosine kinase inhibitors, similar to literature reports. In a study published by Bulusu *et al*. [11], 6 out of 15 patients were treated with tyrosine kinase inhibitors. Only one patient had a partial response lasting < 3 months with imatinib. No durable responses were seen with imatinib, sunitinib, or regorafenib. All five patients with metastatic disease died within 1 year of diagnosis. In a phase II study conducted by Schoffski [15], the efficacy of cabozantinib at 60 mg/day was established. Fifty patients with metastatic GIST were included in the study. Disease control was achieved in 80% of patients. Among them, 14% had partial response and 60% had stable disease. The median time to progression was 6 months. We used cabozantinib in our patient after disease progression with imatinib and sunitinib. However, the disease progressed within 4 months of therapy.

## Conclusion

GIST in a patient with NF1 can occur in young adults and can have atypical features such as unifocal presentation, arising from the stomach without involvement of other parts of the small intestine, and a very rapidly progressive clinical course. Patients with wild-type GIST, especially when it occurs at younger age, should be thoroughly examined for neurocutaneous markers and complete family history, and evaluated accordingly.

## Data Availability

Not applicable.

## Abbreviations

NF1: Neurofibromatosis type 1
GIST: Gastrointestinal stromal tumor
TKI: Tyrosine kinase inhibitor
PDGFRA: Platelet-derived growth factor receptor alpha
SDH: Succinate dehydrogenase
RTK: Receptor tyrosine kinase
